# Case Report of a patient with Madelung’s disease combined with alcoholic liver disease and liver cirrhosis

**DOI:** 10.3389/fmed.20251695921

**Published:** 2025-11-19

**Authors:** Ziming Gu, Yi Hao, Junwei Duan, Keqi Zhao

**Affiliations:** 1 Department of Changchun University of Traditional Chinese Medicine, Jilin, China; 2 Department of the First Clinical Hospital of Jilin Academy of Traditional Chinese Medicine, Jilin, China

**Keywords:** Madelung’s disease, liver cirrhosis, alcohol consumption, metabolic disorder, case report

## Abstract

**Background:**

Madelung’s disease (MD) is a rare disorder of fat metabolism, with symptoms often characterized by the appearance of chronic accumulations of abnormal adipose tissue in areas such as the neck and neckline, upper back and chest. Nowadays, its pathogenesis is still unknown, and some scholars have suggested that its etiology is closely related to alcohol consumption, and that it is associated with a variety of metabolic diseases such as liver disease, hypertension, diabetes mellitus, dyslipidemia, and peripheral neurological damage. Currently, there are very few reported cases of Madelung’s disease complicated by alcoholic liver disease and liver cirrhosis (LC), making this case worthy of reporting.

**Case summaries:**

This article reports on a 60-year-old man, the main point of view “The patient has experienced edema in both lower limbs for 2 weeks, accompanied by a long history of alcohol consumption.” Clinical examination showed subcutaneous swelling of the neck, which has been clearly present for three years. The diagnosis of Madelung’s disease combined with alcoholic liver disease and liver cirrhosis was made based on laboratory tests of liver function, ultrasound of the neck vessels, ultrasound of the superficial tissues of the neck, and CT of the abdomen. The patient has not exhibited restricted neck mobility or symptoms of tracheoesophageal fat syndrome, which affects swallowing and breathing, and refused neck surgery treatment and received hepatoprotective therapy and traditional agents. After 14 days and two cycles of Chinese medicine, edema subsided, and the size of the subcutaneous mass remained stable.

**Results:**

Madelung’s disease is often underdiagnosed and misdiagnosed because of its low prevalence. The purpose of this article is to illustrate the need to be vigilant in clinical encounters with patients with Madelung’s disease combined with alcoholic liver disease and liver cirrhosis, to achieve early intervention and treatment, and to prevent complications. The etiology of this disease remains unclear. Further research into its pathophysiological mechanisms should be pursued, opening new avenues for therapeutic investigations.

## Introduction

Madelung’s disease (MD), also known as multiple symmetrical lipomatosis (MSL), was first reported in 1864 by Brodie (1). Orphanet and the National Organization for Rare Disorders (NORD) in the United States have recognized it as a rare disease (ORPHA number: 2398).

In 1888, Madelung summarized 33 cases of neck lipomas and described their clinical features, from which the name MD was derived. MD is a sporadic fat metabolism disorder, and its clinical manifestations mainly include chronic accumulation of adipose tissue in the neck, upper back and chest. To date, approximately 600 cases have been reported in the domestic and international literature, but there have been no reports of MD patients complicated with liver cirrhosis (LC). In this article, we report a patient with a history of perennial alcohol consumption and a diagnosis of Madelung’s disease combined with cirrhosis of the liver on examination. The most common complication of MD is chronic alcoholic liver disease. However, this report suggests that when we encounter MD with concomitant liver disease, it is important to intervene as early as possible for prevention to avoid cirrhosis and to perform regular liver function tests in patients with MD.

## Case presentation

**FIGURE 1:**
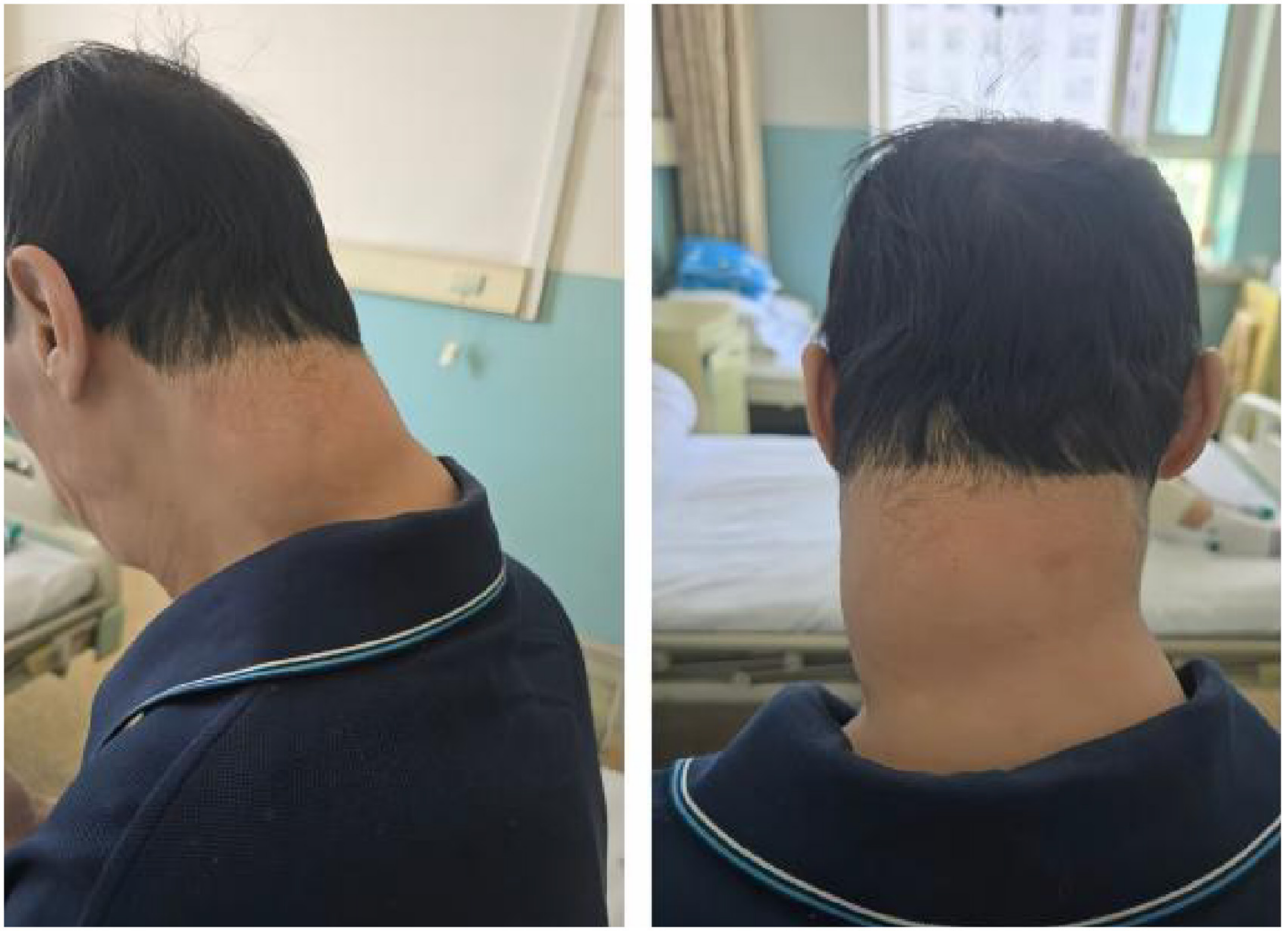
Cervical neck-like mass in the cervical neck.

A 60-year-old male patient presented to the outpatient clinic with bilateral lower extremity edema lasting 2 weeks. Symptoms include edema of both lower extremities, significant symptoms of weakness, gums that bleed easily, and 3 years prior, a movable soft mass was found in the neck that was not accompanied by pain. The patient was admitted to the hospital with “abnormal liver function and cirrhosis on CT of the whole abdomen.” The patient denied a history of viral hepatitis, had a history of severe alcohol abuse (more than 250 milliliters of white wine per day) for more than 30 years, had increased alcohol consumption in the past month, had no family history of similar illnesses, and had no history of specific medication use. The patient was thin, had a BMI of 20.14 kg/m^2^, and had moderate swelling in both lower extremities. The diffuse elevated mass in the cervico-occipital region was soft, movable, and without tenderness, with clear borders (Figure 1).

Physical examination revealed abdominal tenderness, the liver and spleen were not palpable under the ribs, and mobile turbidities were negative; yellow staining of the skin and sclera, and no spider nevus were observed. Flutter tremor was absent. Neurological examination was unremarkable, and neuropsychological testing was normal, with no cognitive impairment. West-Haven Grade 0, the patient did not exhibit clinical manifestations of hepatic encephalopathy, so blood ammonia testing was not performed. Laboratory examination revealed that the patient’s liver function was abnormal, and tests for viral hepatitis-associated antigens and antibodies to screen and identify other factors that may contribute to liver injury were performed, all of which returned to normal. Table 1 below shows the abnormal reference values of the patient’s laboratory tests for liver function.

**TABLE 1:** Abnormal reference values for liver function in patients**’** laboratory tests.

Laboratory test	Result	Normal reference value
Alanine aminotransferase/ALT	33.13	5–40 U/L
Aspartate aminotransferase/AST	80.75↑	8–40 U/L
Alkaline phosphatase/ALP	111.72	30–120 U/L
γ-Glutamyl transpeptidase/GGT	88.99↑	10–47 U/L
Choline esterase/CHE	2065↓	4,000–11,000 U/L
Total bile acids/TBA	18.23↑	≤ 9.67 μmol/L
Total bilirubin/TBIL	56.01↑	5–21 μmol/L
Direct bilirubin/DBIL	25.93↑	≤ 3.4 μmol/L
Indirect bilirubin/IBIL	30.08↑	1.6–21 μmol/L
Total protein/TP	73.60	62–83 g/L
Albumin/ALB	29.81↓	35–52 g/L
Globulin/GLB	43.79↑	20–35 g/L
A/G	0.68↓	1.2–2.5
Prothrombin time/PT	19.70↑	9–13 s
Mean corpuscular volume/MCV	114.0↑	86–100 fL
Serum creatinine/SCr	82.9	35–120 μmol/L

**FIGURE 2:**
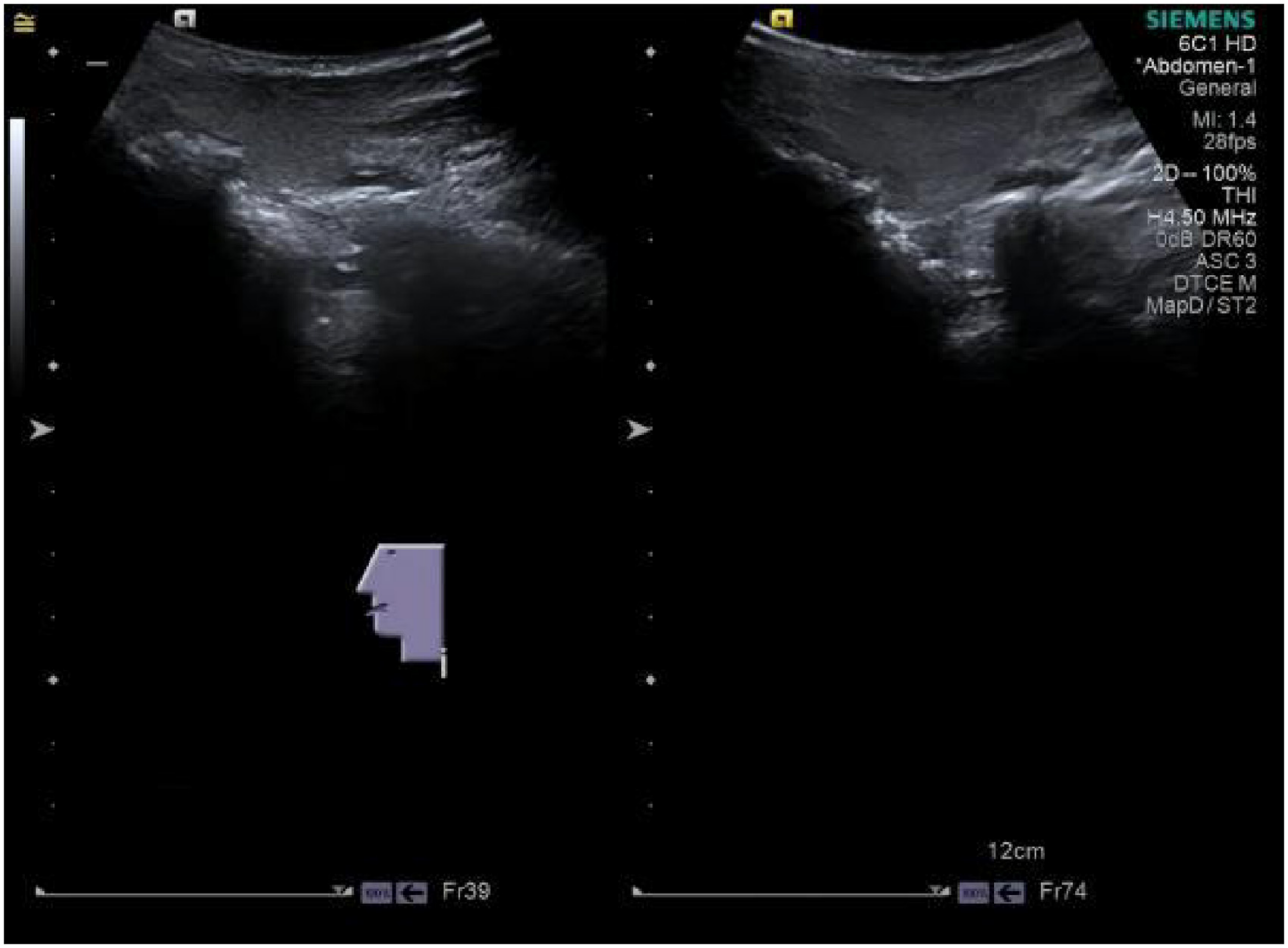
Patient parotid ultrasound.

**FIGURE 3:**
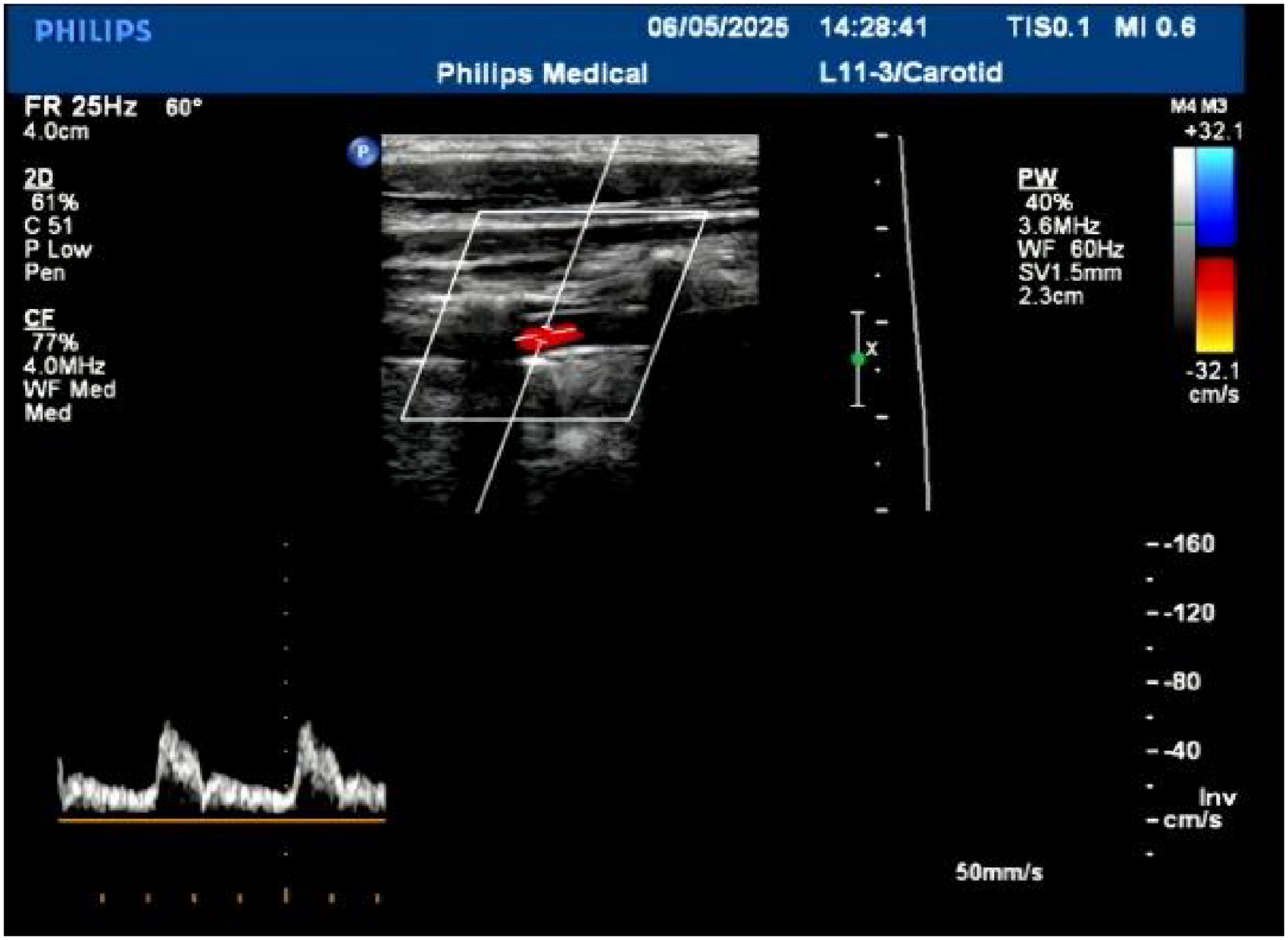
Neck vascular ultrasound.

**FIGURE 4:**
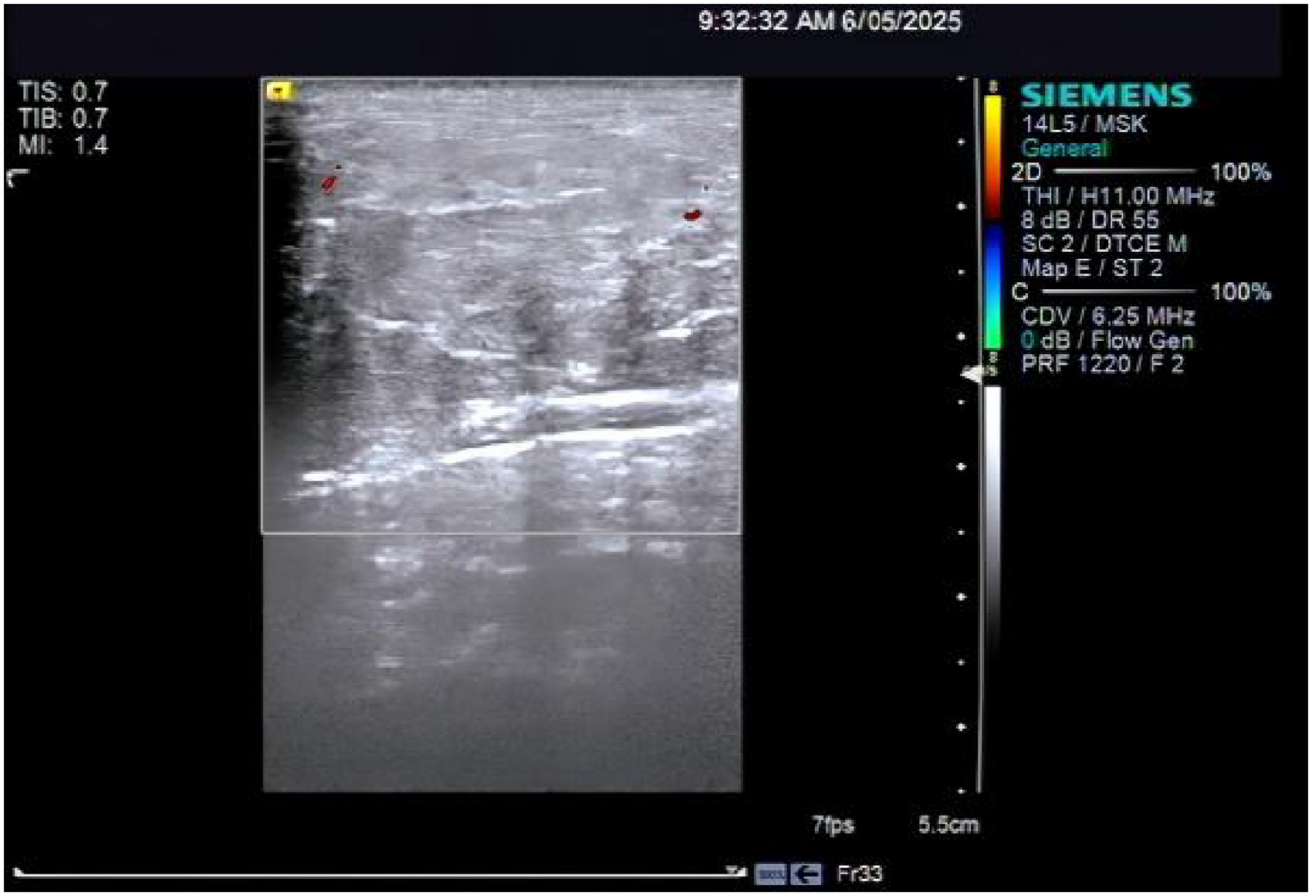
Ultrasound of the superficial tissues of the neck.

**FIGURE 5:**
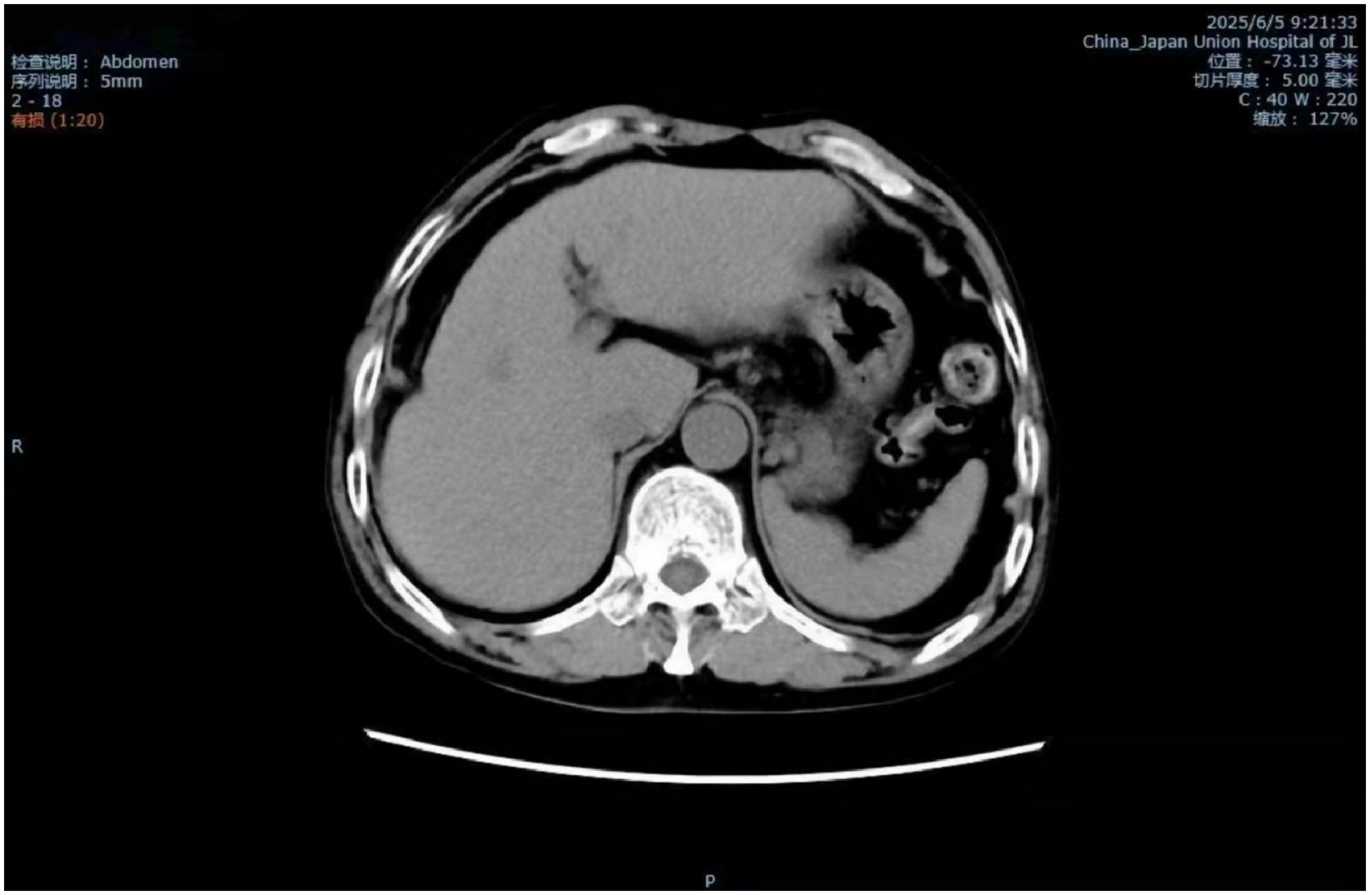
CT scan of the whole abdomen.

The patient’s parotid ultrasound (Figure 2) revealed normal-sized parotid glands bilaterally with no space-occupying lesions, and CDFI revealed scattered punctate blood flow signals visible within the parotid glands. Neck vascular ultrasound as well as superficial neck tissue ultrasound suggested isoechoic soft tissue layers in the posterior neck, suggesting the possibility of a lipoma (Figures 3, 4). The patient was a male with a significant abnormal neck mass on physical examination and a diagnosis of type I Madelung’s disease as suggested by relevant imaging (cervical vascular ultrasound and superficial tissue ultrasound). The abdominal CT scan did not reveal any abdominal fluid accumulation and poorly smooth edges of the liver but no dilatation of the intrahepatic or extrahepatic bile ducts, no splenomegaly, and no dilatation of the portal vein (Figure 5). In conjunction with the patient’s long history of alcohol consumption, imaging findings were suggestive of cirrhosis. The patient consumes over 40 g of alcohol daily, presenting with elevated AST, ALT, GGT, total bilirubin, prothrombin time, and mean corpuscular volume (MCV), with AST/ALT > 2. Imaging reveals diffuse hepatic density reduction and a liver-to-spleen CT value ratio ≤ 1. The diagnosis is alcoholic liver disease (2).

According to the Child-Pugh classification, the patient has a score of 10, corresponding to Class C, indicating severe decompensated cirrhosis. The prognosis may be poor, necessitating heightened vigilance for the development of complications associated with liver cirrhosis.

Clinicians should remain vigilant regarding complications associated with cirrhosis, particularly in patients presenting with ascites. During management, careful differential diagnosis must be applied to distinguish spontaneous bacterial peritonitis from hepatorenal syndrome. In addition, cirrhosis is often accompanied by multiple concurrent complications, all of which require clinical differentiation by physicians. Although this patient has cirrhosis, CT imaging shows no portal vein dilatation. Consequently, symptoms typically associated with portal hypertension—such as esophageal or gastric variceal bleeding or ascites—are absent. Additionally, the patient has a normal temperature, no fever, no abdominal pain, no ascites, and physical examination reveals no abdominal tenderness or rebound tenderness. Spontaneous bacterial peritonitis is therefore not considered. Complications associated with hepatic insufficiency, including hepatic encephalopathy, jaundice, and coagulation disorders, were not evident in the described physical examination or laboratory findings. Additionally, creatinine levels were not elevated, urine output was not reduced, and there were no signs of renal dysfunction or organic changes. Beyond clinical presentation and physical examination, the differential diagnosis of cirrhosis may also involve abdominal paracentesis, gastroscopy, imaging studies, and laboratory tests.

The patient was eventually diagnosed with Type I Madelung’s disease complicated by alcoholic liver disease and cirrhosis. The patient did not experience neck mobility or fat compression of the tracheoesophagus, which affects swallowing and breathing, and refused surgical treatment of the neck; therefore, he did not undergo surgical treatment or pathological examination and received the treatment plan involving protection of liver function and Traditional Chinese Medicine. Traditional Chinese medicine employs dandelion as a single-herb *T. officinale*, with 15 g of dandelion boiled in 400 mL of water daily for oral administration. Hepatoprotective therapy involves administering 75 mg of disulfiram daily, divided into three doses. Doctors advise patients to abstain from alcohol consumption in the future to control the progression of Madelung’s disease and alcoholic fatty liver disease. A light diet should be maintained, with judicious salt intake and controlled fluid consumption to prevent ascites formation. Bed rest should be prioritized wherever possible to alleviate hepatic burden and reduce complication risks. However, prolonged bed rest may induce muscle wasting. Appropriate exercise can also prevent lipoma growth, while protein supplementation and increased branched-chain amino acid (BCAA) intake (combination therapy) should be provided (3).

Upon discharge, the patient’s liver function was satisfactory. While gamma-glutamyl transpeptidase (GGT) levels remained elevated at 62.22 U/L, all other laboratory parameters were within normal ranges. However, no significant changes were observed in the fatty deposits in the neck. It is recommended that the patient undergo abdominal ultrasound or CT scans every 1–3 months and regularly monitor the growth of the neck mass.

## Discussion

Madelung’s disease is a rare disease that is more common in the Mediterranean region and less prevalent in Asia. It is characterized by the chronic accumulation of abnormal fatty tissue in the neck, upper back and chest (4). According to reported cases, the disease occurs in middle-aged and older men, with a lower proportion of women, and all have a long history of alcohol consumption (5). The case report in this article involves a 60-year-old male patient with a history of alcohol consumption for more than 30 years (more than 250 mL per day).

MD was found to be associated with a variety of factors after 600 cases, including chronic alcohol consumption, metabolic disorders (such as hypertension, hyperlipidemia, diabetes mellitus, peripheral neurological injuries, or hyperuricemia), liver disease, hypothyroidism, and renal tubular acidosis (4, 6, 7).

**FIGURE 6:**
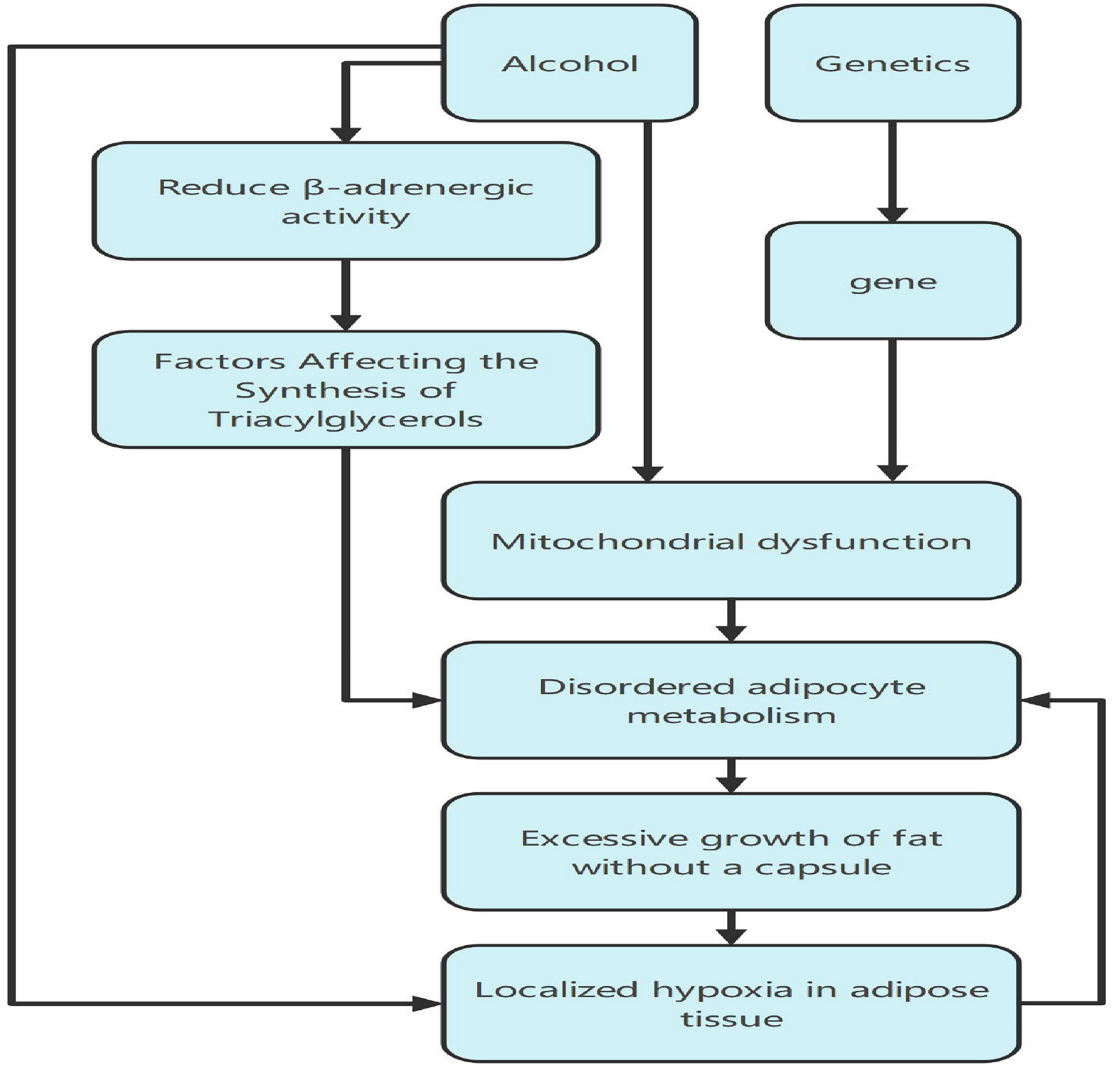
Mechanism of MD.

The mechanism of the disease is still unclear, but Hu Bo et al. reviewed current research progress on the pathogenesis of MD, summarizing its histological features and genetic alterations. However, multiple omics studies on MD have yet to yield a unified conclusion (8). Many cases have been analyzed, and 85% of the patients suffering from the disease have a history of alcoholism for more than 10 years (9, 10). Additionally, familial inheritance may occur due to alterations in autosomal genes, with some cases potentially being autosomal dominant (11). Chronic alcohol abuse leads to functional defects in brown adipocytes (12, 13), mutations in mitochondria and chromosomes, and premature oxidative (14). Chronic alcohol consumption also causes a decrease in the number and activity of β-adrenergic receptors required for lipolysis, thus affecting triacylglycerol synthesis (15), as well as impaired catabolism and metabolism of brown adipose tissue under the influence of catecholamines, characterized by unencapsulated hypertrophic growth and localized hypoxia within adipose tissue, which ultimately promotes the development of lipomas (13) and abnormalities of fat deposition (9, 12). Alterations in autosomal genes frequently involve mutations in the MNF2 and LIPE genes. The enzyme encoded by the MNF2 gene is located in the mitochondrial outer membrane and participates in mitochondrial fusion and cellular energy metabolism processes (16); the LIPE gene encodes hormone-sensitive lipase, a key enzyme in triglyceride metabolism, primarily expressed in adipocytes (17). Notably, although men are more likely to develop Madelung’s disease based on cases, mitochondrial DNA mutations and nuclear genes associated with mitochondrial maintenance were not detected (Figure 6).

**FIGURE 7:**
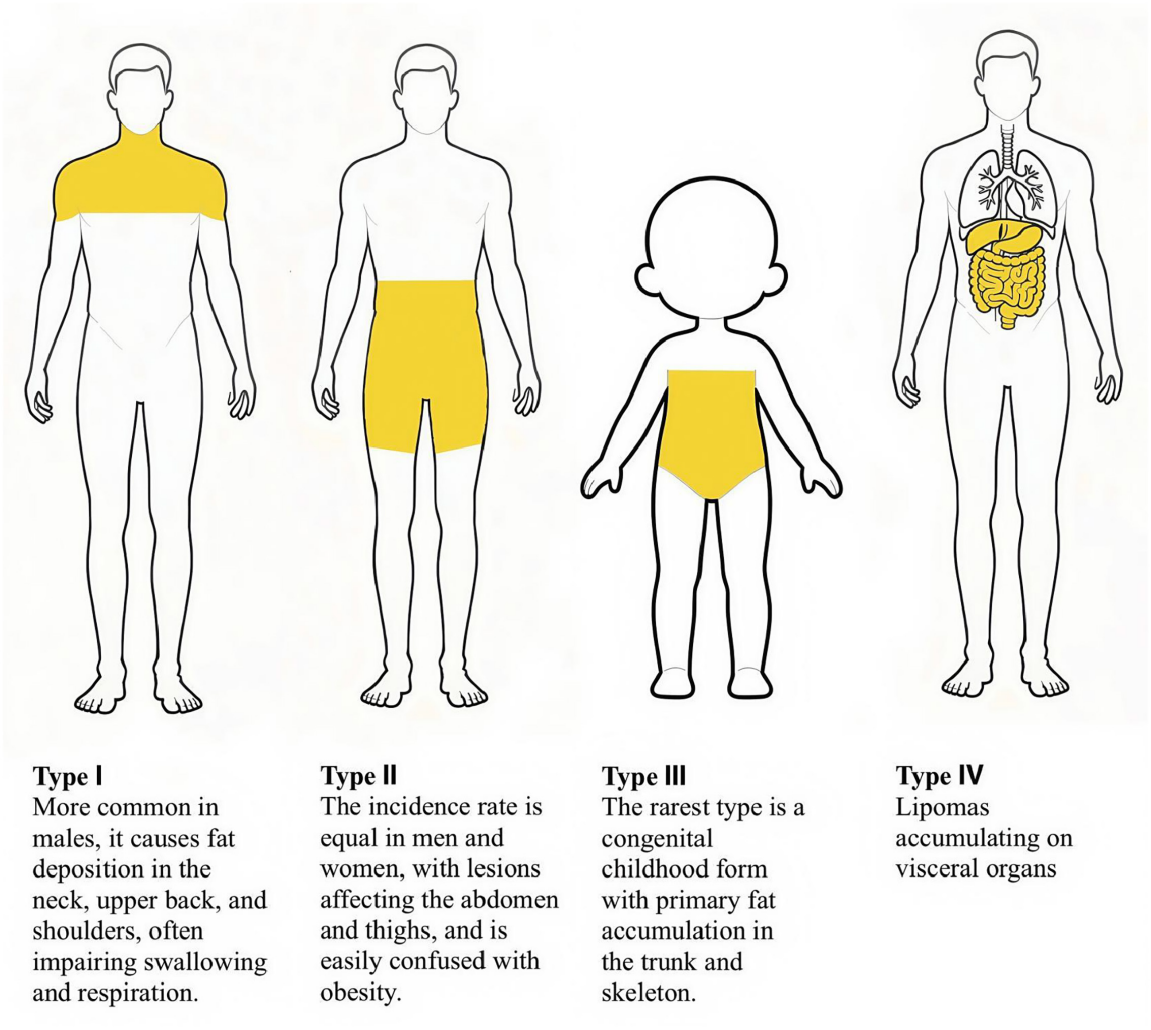
Four types of MD.

Currently, the diagnosis of MD relies on imaging and clinical presentation, with biopsy if necessary. Given that the patient did not cause discomfort and refused to undergo pathological examination, the pathological specimen of MD is generally considered to contain many smaller adipocytes, fibrous connective tissue, and vascular hyperplasia in the interstitium (18). Based on the clinical features and distribution of their masses, MDs can be classified into four types (19–22) (Figure 7):

Type I lesions are more common in men than in women. The lesions manifest as symmetrical bulges on the upper part of the body, with swelling diffusely distributed in the neck, neck, upper back, shoulder, and proximal part of the upper limbs, presenting the characteristic manifestations of “horse’s neck” or “cow’s neck.” Due to its common site of occurrence, Type I MD may be easily confused with neck tumors. Key points for differential diagnosis require a comprehensive patient assessment, including medical history, physical examination, imaging studies to evaluate the location, size, and tissue compression of the fat deposit, and histological analysis. There are also a few cases of fat accumulation in the breasts. There are also a few cases of fat accumulation in the breasts. This type of MD is often accompanied by compression of the cervical blood vessels, trachea, laryngeal cavity, and brachial plexus nerves, leading to head and neck swelling, **dyspnea**, venous stasis, and brachial plexus neuropathy (19, 23).

There have also been case reports of fat accumulation leading to hypertrophy of the tongue, which interferes with swallowing and breathing, creating obstructive sleep apnea hypoventilation syndrome (23).

Type II MD has a similar prevalence in both men and women and is also known as the “pseudoathlete type,” It mainly involves fat accumulation in the abdomen and thighs. However, the neck and upper trunk regions are not affected. This type is similar to simple obesity and is often confused.

Type III MD is the rarest type, is congenital, has predominantly skeletal trunk lesions, and occurs most often in children.

Hippolyte Dupuis proposed a fourth type of MD, which is characterized by lipomas affecting internal organs, such as pancreatic lipomas and adrenal myelolipomas (22).

Patients may present with one of the above types or multiple types at the same time, and this case supports the diagnosis of type I Madelung’s disease. In addition, the left neck and left supraclavicular fossa in this patient had more fatty foci than the contralateral lesion, which is also consistent with fat accumulation in patients with Madelung’s disease and should not be considered malignant. Distinguishing a neck lipoma from other neck tumors, especially malignant ones, is also a process of differentiating between “benign and malignant” and “safe and dangerous.”

The most intuitive initial clinical judgment is often made by physical examination, where the doctor assesses the nature of the mass by palpation; lipomas are softer in texture, well-mobile and slow-growing, while malignant tumors are firm, fixed in mobility and fast-growing. Malignant tumors are often accompanied by other symptoms such as enlarged lymph nodes elsewhere (24), voice changes, nasopharyngeal symptoms or systemic symptoms.

Ultrasound examination serves as the primary investigation for confirming the adipose nature and benign characteristics of subcutaneous nodules (17). Its advantages include being non-invasive and cost-effective. It can clearly distinguish the nature of the mass (solid or cystic) and provide an initial assessment of its blood supply, borders, and relationship with surrounding tissues. Ultrasound is highly valuable for differentiating lipomas (typically appearing as well-defined masses with homogeneous echogenicity) from other tumors. CT and MRI provide clearer depiction of the precise location, size, and relationship of the mass to major surrounding vessels and nerves, as well as the status of cervical lymph nodes. These modalities are particularly essential for deep-seated or complex masses.

Needle biopsy is the gold standard for distinguishing Madelung’s disease from malignant tumors. Adhering to non-maleficence, biopsy is generally not performed in patients whose quality of life remains unaffected, with diagnosis instead relying on a comprehensive assessment. Fine-needle aspiration (FNA) involves using a thin needle to extract a small sample of cells from a mass for pathological examination. This minimally invasive procedure is a crucial method for distinguishing between benign and malignant conditions. If FNA fails to provide a definitive diagnosis or if malignancy is strongly suspected, the entire mass may be surgically removed for pathological examination, serving both diagnostic and therapeutic purposes.

Pathological examination can isolate adipose-derived stem cells (ASC) from MD patients. MD adipose tissue is characterized by smaller adipocytes with atypical nuclei, and thicker, more regular intercellular septa (7). Furthermore, it is essential to differentiate polymyositis from conditions such as obesity, Cushing’s syndrome, lipomatosis vasculorum, encapsulated lipoma, neurofibromatosis, myxoid liposarcoma, lymphoma, and lipodystrophy syndromes.

Currently, the main treatment for MD is still surgery, including excision and liposuction, with excision being the preferred method in most cases and liposuction being used mainly for smaller fat masses. However, fat is diffusely distributed with normal body tissues and muscles, so it is difficult to remove all of it, and there is still a high recurrence rate after surgery. Although surgery can restore the body’s normal form and improve symptoms such as swallowing and breathing, the most crucial treatment to prevent recurrence is still abstinence from alcohol, which is the fundamental cause of Madelung’s disease. Controlling alcohol intake is an important measure to prevent recurrence (25).

In the absence of significant life impediments, this would be a better treatment if an effective medication could be found, but medicated lipolysis is prone to recurrence and localized fibrosis and adhesions, and recurrence poses difficulties for the next treatment. Commonly used therapeutic drugs to restore mitochondrial function include salbutamol, vitamins C and E, coenzyme Q10, and L-carnitine.

The pathophysiology of MD remains poorly understood, with its mechanisms still unclear. Current treatments can only alleviate symptoms symptomatically, and research has tended to focus more on macro-level clinical manifestations. This has resulted in a rather simplistic characterization of the disease’s features, necessitating deeper investigation in the future. Given the relatively rare incidence of MD, existing studies are confined mainly to case series reports and case sharing, with small sample sizes. This may compromise their generalizability and statistical significance. Establishing large-scale biobanks for future studies is increasingly imperative to advance MD research. Although most cases indicate alcohol exerts a specific effect on certain adipocytes, investigations into alcohol-related factors remain scarce. MD frequently presents with pathological features such as inflammation, mitochondrial dysfunction, and oxidative stress. These findings may open new avenues for diagnosis and treatment in this field (26, 27).

In this study, we did not perform surgery on the patient because he had no symptoms affecting his quality of life and was comorbid with cirrhosis. We recommended treatment by abstaining from alcohol, reducing salt, controlling water intake, resting in bed, and taking liver-protecting medications and traditional Chinese medicine. Patients with liver cirrhosis should restrict fluid intake primarily to control sodium and water retention, thereby managing ascites and edema, and correcting hyponatraemia. The key lies in “restricting sodium” rather than merely “restricting water.” Daily salt intake for cirrhosis patients should not exceed 5–6.5 g (28). Not all patients require strict water restriction. Only when hyponatraemia occurs will physicians impose strict limits on total daily fluid intake. Given the varying severity of each patient’s condition, doctors formulate individualized plans to prevent further sodium depletion, which could trigger serious complications such as cerebral edema. Bed rest holds significant therapeutic value for patients with decompensated cirrhosis, particularly those with substantial ascites. This position alleviates gravitational effects, effectively increasing venous return to the heart and renal blood flow. It improves glomerular filtration rate, promotes urinary sodium excretion, and aids in the resolution of ascites and edema. Rest also helps reduce portal vein pressure, thereby lowering the risk of bleeding. *T. officinale* has long been employed as a primary constituent in various hepatoprotective herbal formulations. Research has analyzed published studies examining dandelion’s effects in animal models and its potential applications in liver disease. Taraxasterol, one of dandelion’s principal bioactive compounds, modulates inflammatory and oxidative stress pathways, thereby preventing liver damage and ascites formation (29). Furthermore, reports indicate significant therapeutic efficacy in reducing lipid accumulation associated with metabolic dysfunction-associated fatty liver disease, alongside marked decreases in body weight, liver mass, triglycerides, and total cholesterol. From the treatment of this case, Traditional Chinese medicine has demonstrated efficacy in protecting the liver, reducing lipid accumulation, and preventing ascites; however, its effectiveness requires validation through prolonged clinical trials and observation. Herbal medicine has shown promising efficacy in eliminating edema and reducing peritoneal fluid, but more clinical trials are needed to validate this further. As MD is often associated with liver damage, liver function tests should be given top priority during follow-up, and abdominal ultrasound or CT should be performed once every 1–3 months to monitor the build-up of lumps in the neck.

## Conclusion

Owing to the very low prevalence of MD in Asia, there is still underdiagnosis and misdiagnosis, and there are only isolated cases of patients whose liver disease has progressed to cirrhosis with comorbid Madelung’s disease. This report aims to provide clinical assistance in diagnosis and treatment and to maximize therapeutic outcomes. The disease is caused by years of alcohol consumption, which can lead to liver damage; thus, when such patients are present in the clinic, the attention and follow-up of liver function indices becomes a top priority.

## Data Availability

The original contributions presented in this study are included in the article/supplementary material, further inquiries can be directed to the corresponding author.
